# Editorial: Minimally invasive vascular surgery

**DOI:** 10.3389/fsurg.2024.1362571

**Published:** 2024-01-15

**Authors:** George Galyfos, Athanasios Katsargyris, Dimitrios Liakopoulos, Konstantinos Filis

**Affiliations:** ^1^ Vascular Unit, First Propedeutic Department of Surgery, Hippocration Hospital, National and Kapodistrian University of Athens, Athens, Greece; ^2^ Second Department of Vascular Surgery, Laikon Hospital, National and Kapodistrian University of Athens, Athens, Greece; ^3^ Vascular Unit, Department of Surgery, Nikaia General Hospital, Athens, Greece

**Keywords:** minimally invasive vascular surgery, endovascular, hybrid vascular procedures, robot-assisted vascular surgery, innovation

**Editorial on the Research Topic** Minimally invasive vascular surgery

## Editorial

Vascular surgery has been evolved during the last two decades significantly, especially after the introduction of endovascular and less invasive techniques. The main aim of performing minimally invasive vascular procedures has always been to minimize perioperative mortality and morbidity as well as to accelerate rehabilitation and return to everyday activities for the patients. Such techniques may include endovascular or hybrid procedures, laparoscopic approaches as well as robot-assisted techniques (1).

Endovascular interventions have been broadly used for treating pathologies that otherwise would be difficult or unthoughtful to treat. For years, the main strategy for treating deep vein thrombosis (DVT) has been the conservative therapy using anticoagulants and compression stockings. However, the evolution of endovascular tools has allowed physicians to treat nowadays proximal DVTs in order to reduce the risk for pulmonary embolism (PE) and post-thrombotic syndrome (2). Zhang et al. have prospectively evaluated the effect of endovascular treatment of DVT as far as the risk for PE is considered. After placing a retrievable inferior vena cava filter (RIVCF) in a cohort of patients, they concluded that patients treated conservatively had a higher risk for RIVCF embolism compared to patients treated with thrombolysis/thrombectomy +/− angioplasty for DVT. For selected cases, such a minimally invasive strategy would be therefore both safe and effective.

Endovascular therapy has also contributed significantly to the management of vascular trauma, offering a great benefit especially for multi-trauma patients (3). Bayona et al. have described an interesting case of a patient with penetrating trauma of the thoracic aorta as well as injury of other intra-abdominal structures. The patient was primarily treated for the aortic rupture with the placement of a covered stent, and then the abdominal injuries were addressed with laparotomy after the patient had been stabilized. This report underlines the contribution of minimally invasive techniques to such cases where traditional open approaches would be difficult or catastrophic. Chaves et al. have also described a case series of patients with arterial injury due to central venous catheterization that were treated using vascular closure devices. This minimally invasive technique could help avoid a thoracotomy or sternotomy that are associated with a higher mortality and morbidity, especially in patients with severe comorbidities.

Hybrid techniques also combine the advantages of both open and endovascular approaches offering the possibility to treat complex cases without the need of extended open procedures (1). Park et al. describe such a case suffering from Ehlers-Danlos syndrome that presented with a dissecting aneurysm of the right iliac artery and an arteriovenous fistula between the left internal iliac artery and common iliac vein. The patient was treated successfully without any major complication. Connective tissue diseases comprise a challenge as far as the treatment of associated vascular complications is concerned. Open repairs are quite demanding considering the dissection and handling of the vessels while endovascular repairs are associated with high bleeding risk at the access sites (4). Therefore, hybrid techniques could be a reasonable solution for such high-risk patients.

Minimally invasive techniques can be also utilized not only for treatment but also for prevention of complications. He et al. have reported the results of a comparative study where they evaluated the effect of far infrared therapy on major outcomes of arteriovenous fistulas among patients with chronic renal disease. It was shown that such a novel technique can improve the function and prolong the longevity of the fistulas. Such techniques could therefore help reduce the risk for late occlusions or secondary interventions.

Besides the aforementioned achievements, several other studies have been published showing the progress in minimally invasive vascular surgery (MIVS) (1). MIVS also includes techniques that refer to: the endovascular repair of complex aortic aneurysms using fenestrated endografts, the endovascular therapy of peripheral artery disease using novel atherectomy or reentry devices, robot-assisted vascular or endovascular procedures, hybrid repair such as transcarotid stenting for carotid artery stenosis and others (1, 5, 6). Besides the well-known advantages, physicians should also focus on the limitation of risks associated with the aforementioned MIVS procedures such as the risk for spinal cord ischemia, endoleaks, fracture or occlusion of stents or branches, migration of grafts or stents, reduced long-term patency compared to open surgery, contrast-mediated renal insufficiency and others, in order to maximize their benefit (7, 8).

In conclusion, MIVS seems to be the most appropriate—and in some cases the only available—therapeutic strategy for treating vascular pathologies with low perioperative risks. However, these promising results should be further verified with larger comparative studies in order to evaluate both early and late outcomes.

## References

[1] Galyfos G Liakopoulos D Sigala F Filis K. New paradigms in minimally-invasive vascular surgery. Expert Rev Cardiovasc Ther. (2022) 20:207–14. 10.1080/14779072.2022.2058492 35341434

[2] Avgerinos ED Saadeddin Z Abou Ali AN Pandya Y Hager E Singh M Outcomes and predictors of failure of iliac vein stenting after catheter-directed thrombolysis for acute iliofemoral thrombosis. J Vasc Surg Venous Lymphat Disord. (2019) 7:153–61. 10.1016/j.jvsv.2018.08.014 30660580

[3] Filis K Sigala F Stamatina T Georgia D Zografos G Galyfos G. Iatrogenic vascular injuries of the abdomen and pelvis: the experience at a Hellenic university hospital. Vasc Endovascular Surg. (2019) 53:541–6. 10.1177/1538574419858809 31248345

[4] Brooke BS Arnaoutakis G McDonnell NB Black JH 3rd. Contemporary management of vascular complications associated with Ehlers-Danlos syndrome. J Vasc Surg. (2010) 51:131–9. 10.1016/j.jvs.2009.08.019 19879095 PMC6042287

[5] Katsargyris A Marques de Marino P Hasemaki N Nagel S Botos B Wilhelm M Editor’s choice—single centre midterm experience with primary fenestrated endovascular aortic aneurysm repair for short neck, juxtarenal, and suprarenal aneurysms. Eur J Vasc Endovasc Surg. (2023) 66:160–6. 10.1016/j.ejvs.2023.02.069 36842460

[6] Galyfos GC Tsoutsas I Konstantopoulos T Galanopoulos G Sigala F Filis K Editor’s choice—early and late outcomes after transcarotid revascularisation for internal carotid artery stenosis: a systematic review and meta-analysis. Eur J Vasc Endovasc Surg. (2021) 61:725–38. 10.1016/j.ejvs.2021.01.039 33674158

[7] Westin GG Rockman CB Sadek M Ramkhelawon B Cambria MR Silvestro M Increased ischemic complications in fenestrated and branched endovascular abdominal aortic repair compared with standard endovascular aortic repair. J Vasc Surg. (2020) 72:36–43. 10.1016/j.jvs.2019.09.044 32081484

[8] Aru RG Tyagi SC. Endovascular treatment of femoropopliteal arterial occlusive disease: current techniques and limitations. Semin Vasc Surg. (2022) 35:180–9. 10.1053/j.semvascsurg.2022.04.010 35672108

